# Sinoatrial Reentry Tachycardia: A Review

**Published:** 2003-07-01

**Authors:** TA Simmers, N Sreeram

**Affiliations:** * Department of Cardiology, Academic Medical Centre, Amsterdam, The Netherlands; † Dept. of pediatric cardiology, University Hospital of Cologne, Germany

## History and Electrophysiology

The concept of reentry within the sinus node is by no means new. In their 1943 report, Barker and co-workers postulated that “…a circus rhythm could be accommodated in auricular muscle and in one of the specialized nodes at known rates of conduction and with cycle lengths such as occur in paroxysmal tachycardia” [1]. Lack of invasive electrophysiology at that time and subsequent failure to appreciate the heterogeneity of supraventricular arrhythmias, left their astute observation in the realm of conjecture. It was not until 1968 that Han, Malozzi and Moe finally demonstrated the existence of sinoatrial echoes; [2]. In a superfused isolated rabbit right atrial preparation, they examined the response to premature extrastimuli with an 18 electrode grid at the sinus node and surrounding atrial tissue. They found that critically timed extrastimuli led to early re-excitation of the atrium, supposedly due to sinoatrial reentry. Sequential microelectrode measurements of sinus node and atrial transmembrane potentials were performed in an attempt to provide a temporal and anatomical map of the observed phenomenon. Based on findings, the authors concluded that sinoatrial reciprocation was caused by entrance block at one site, slow conduction within the node with disparate refractoriness, and re-excitation of the atrium at the original area of entrance block. They went on to suggest that repetition of this phenomenon may conceivably form the basis for clinically relevant tachycardia. In vivo confirmation of sinoatrial echoes was inferred from work by Childers; [3] and Paulay; [4]. In the former study, programmed electrical stimulation was performed in dogs. Three responses to premature atrial stimuli were noted: complete interpolation (i.e. the subsequent sinus complex was on time), incomplete interpolation (i.e. the subsequent beat was delayed), and sinus echo. In the latter, the first post-extrastimulus complex was electrocardiographically the same as sinus, but earlier than expected. The authors attributed this to sinoatrial reentry, and went on to describe each of the three responses to premature atrial beats in a 70-year-old man in an elegant electrocardiographic deduction of underlying mechanisms. An in vivo canine model was also employed in the report by Paulay et al. While confirming Childers’ findings, Paulay offered two additional pieces of evidence implicating the sinus node/perinodal area: firstly, timing of the echo was independent of stimulation site (they performed pacing at the sinus node, Bachmann’s bundle, right and left atrial appendages and low atrial septum), and secondly echoes were eliminated by crushing the sinus node. As has long been the case for AV nodal reentry tachycardia, it is apparent that considerable discussion also revolves around the issue of whether sinoatrial echoes do or do not involve perinodal atrial tissue as well as the sinus node itself. In 1979, Allessie and Bonke published work suggesting confinement of the re-entrant circuit to the node per se [5]. The experimental set-up was similar to earlier work by Han [2], with the significant difference that resolution of the measuring electrodes was superior: 32 unipolar surface atrial electrograms were recorded during initiation of sinoatrial echoes as opposed to the 18 used by Han. In addition, up to 130 transmembrane sinus node potentials were recorded sequentially in cases of sustained reciprocation. These detailed observations led to the conclusion that sinus node reentry was confined to a circuitous pathway in an extremely small area (1-2mm) with low conduction velocities (2.5cm/s) within the node itself. It is of note that the same authors pointed out the inability to induce sustained arrhythmia in this model, and later concluded that sinoatrial reentry tachycardia (SART) may not be feasible without border zone atrial tissue. Given the limitations inherent to in vivo study in man, it may be fair to continue to use “sinus node reentry” and “sinoatrial reentry” synonymously for the time being. In the wake of in vivo studies in dogs, and reports of sinus node reentry in man by Paulay [6] and Childers [3], Narula first described sustained SART in two patients in 1974 [7]. Out of 300 patients undergoing electrophysiologic study, he observed sinus node reentry beats in 20, and sustained tachycardia in two. The criteria for the diagnosis of SART proposed by Narula are still valid: 1. atrial activation and P-wave morphology are the same or highly similar to sinus rhythm, with activation from high to low right atrium, 2. the arrhythmia is inducible with atrial extrastimuli at specific coupling intervals, independent of AV nodal conduction intervals and site of stimulation, and 3. the arrhythmia can be terminated by atrial stimuli. SART is thus, by definition, a paroxysmal arrhythmia.

## Clinical Presentation

Symptoms of SART vary widely. Patients may present with paroxysmal palpitations, dyspnoea, dizziness, (near-) syncope, chest discomfort and other symptoms [8]. Given the electrocardiographic similarity to sinus tachycardia and the on average lower heart rates than, for example, circus movement using a concealed bypass [9], a degree of under-diagnosis is likely. Patient’s symptoms can in fact be so reminiscent of anxiety disorders that a psychiatric diagnosis is initially entertained and referral to a cardiologist unnecessarily delayed. Again, electrocardiography is usually typical, with sinus rhythm morphology P waves and RP:PR ratio of greater than one. In one report, however, prolonged AV nodal conduction leading to superposition of the P wave on the preceding T wave or ST segment was shown to cause confusion with a diagnosis of AVNRT in 27% of a cohort of 65 patients undergoing EP study for symptomatic paroxysmal SVT [10]. In general, there are only two serious electrocardiographic differential diagnoses: inappropriate sinus tachycardia, and atrial reentry tachycardia arising near the sinus node [11]. The former differs primarily in its non-paroxysmal character, with an often elevated resting rate and gradual but excessive acceleration as a reaction to even mild exercise; the latter, also being a paroxysmal arrhythmia, can be extremely difficult to differentiate from sinoatrial reentry. Reaction to vagal manoeuvres and adenosine may provide clues to the correct diagnosis, as both tend to terminate sinoatrial but not intraatrial reentry tachycardia. Differentiation between SART, inappropriate sinus tachycardia and intraatrial reentry tachycardia is detailed in Table 1. Reported incidence of SART varies widely, although it is by any standards a relatively uncommon arrhythmia. A study by Wellens and co-workers demonstrated SART in only 1.8% of 379 patients undergoing diagnostic EP study [12]; however, the large proportion of patients included with VT and atrial flutter may cause underestimation of overall prevalence in the general population. At the other end of the spectrum, Gomes et al reported an incidence of 16.9% in a study mentioned earlier [10]; mean age of SART patients was 60 years and all but one had concomitant organic heart disease, which in this case may lead to overestimation of overall prevalence. The finding that SART in adults is associated with a relatively high prevalence of heart disease, be it hypertensive, ischemic or valvular, has been reported by several other authors [3,7,9,13-16], as much as 100% in the 7 patients reported by Wu et al [9]. Interestingly, although numbers are small this does not seem to hold true in the paediatric population. Probably the first case of SART reported in a child, an otherwise healthy 10-year-old, was published by Pahlajani, Miller and Serrato in 1975 [17]. The following year, Gillette reported SART in 5 of 35 children aged below 18 years undergoing diagnostic EP study for symptomatic SVT [18]; three had no organic heart disease, and two an ASD (of which one previously operated). Work by both Garson [19] and Blaufox [20] further underlines the relationship between recent cardiac surgery and SART in children, with 70% in the latter study having recently undergone corrective or palliative surgery mainly for hypoplastic left heart syndrome.

## Treatment

**Figure 1:**
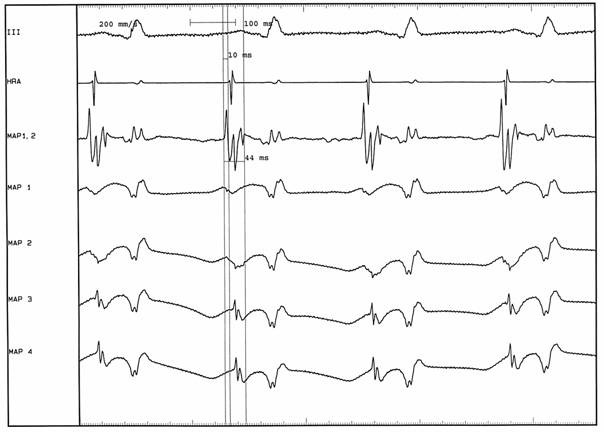
Endocardial recordings during SART in the infant reported by Simmers et al in reference 36; while the local electrograms are less premature relative to the P-wave than in adults, the fragmentation (44ms) and the cranial to caudal activation pattern can be easily appreciated in the unipolar recordings (MAP 1 - MAP 4)

**Figure 2:**
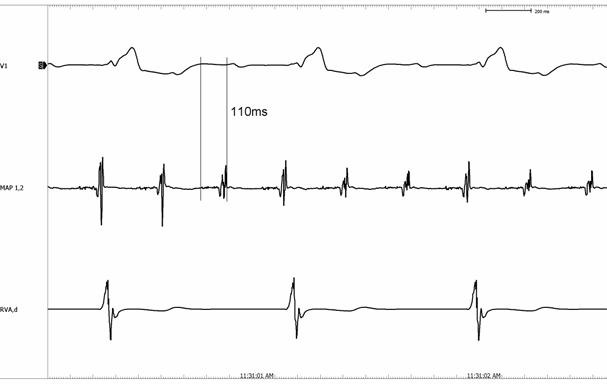
An adult patient, in whom the atrial electrogram at the successful ablation site is markedly more fragmented, and in this case 110ms before the P-wave in the surface ECG. Ablation was subsequently successful at this site

In contrast to inappropriate sinus tachycardia and atrial tachycardias, SART rarely responds well to β-blockers. Digoxin, calcium channel blockers such as verapamil and amiodarone are the drugs of choice [10]. In drug refractory cases, aversion to long-term pharmacotherapy or in the event of severe symptoms, a more curative invasive approach may be warranted. Surgical modalities are no longer preferred since the advent of endocardial catheter ablation, but are nonetheless interesting from an historical viewpoint. In 1984, Yee and co-workers described management of what was probably inappropriate sinus tachycardia by subtotal right atrial exclusion in a 27-year-old woman [21]. This method entailed the amputation of a large part of the right atrium from all surrounding structures, severing the sinus node artery, and subsequently suturing the excluded portion back. This resulted in a stable junctional rhythm and palliation of symptoms in the patient described. More recently, catheter ablation has been demonstrated to be a feasible method with which to isolate the offending sinus node [22,23]. Hendry et al attempted surgical treatment, again of inappropriate sinus tachycardia rather than SART, by simply excising the sinus node area of the right atrium [24]. Of the three patients described, only one was cured; the other two subsequently developed new arrhythmias. Conceptually much closer to present techniques of catheter ablation, Kerr and co-workers described epicardial cryoablation at the site of earliest atrial activation at the superior vena cava - right atrial junction in a 42-year-old woman with SART [25]. This terminated the tachycardia, followed by which a stable atrial escape was seen to originate 3cm inferior and posterolaterally to the ablation site. Additional cryoablation was required at a second surgery 4 days later due to early recurrence, after which the patient remained free of SART during 14 months follow-up. The literature on endocardial radiofrequency catheter ablation for SART is obviously more recent, and also limited to small numbers of cases. Kay [24], Sperry [15], Lesh [16] and Poty [26] all reported 100% acute success of ablation for SART at the site of earliest atrial activation, each in no more than 4 patients. Poty et al describe characteristics of successful ablation sites in slightly more detail, referring to fragmentation of local atrial electrograms as a marker of success. They also used unipolar electrograms for mapping purposes, in contrast to the bipolar mode described by other authors. The advantages of the unipolar recording mode for endocardial mapping have been described elsewhere in detail for a wide variety of arrhythmias [27-33]. In the event of fragmented, multiphasic bipolar electrograms as in the case of SART as reported by Poty and others [26,34-36], one of the principle advantages of the unipolar mode is reliable determination of local activation time. Sanders [34] and Ivanov [35] reported the two largest series of radiofrequency catheter ablation for SART, 10 patients each with a 100% success rate. Sanders et al reiterate the importance of ablation at the site of earliest atrial activation during tachycardia, but go further in identifying specific characteristics of successful versus unsuccessful sites. They found that atrial activation at 35ms or more before onset of the P-wave in the surface ECG and more than 20ms before high right atrium, and fragmented electrograms (they described an average of 87ms) were associated with successful ablation. A wide range of timing has been reported to represent "early" atrial activation, from -20ms [35] to -100ms [15]; however 25-35ms pre-P is usually cited, as in the Sanders report. The youngest patient to undergo ablation for SART to date was a 2-month-old infant reported by Simmers, Sreeram and Wittkampf. While the patient demonstrated all typical electrophysiologic findings of SART, there was markedly less prematurity of local activation relative to the P-wave during tachycarida than usually reported in adults [36]. It is conceivable that this difference was due to the fact that the patient weighed only 3000g. While complications of ablation for SART are rare, two issues deserve attention: firstly, right hemidiaphragmatic paralysis. Due to the close relation between the phrenic nerve and ablation site in some individuals, it is of the utmost importance to perform high output pacing (10mA or more) at any putative ablation site, to identify phrenic stimulation and thus risk of thermal injury on applying radiofrequency current. Secondly, ablation near the junction between right atrium and superior vena cava (SVC) may entail a risk of SVC stenosis. In a recent report on ablation for inappropriate sinus tachycardia by Leonelli and co-workers [37], 3 of the 35 patients developed SVC stricture. Whether this may also apply to SART is unclear; ablation for IST usually requires more ablation lesions than for SART, and most importantly all 3 cases reported by Leonelli had undergone pacemaker implantation or upgrade during the same procedure. Figures 1 and 2 demonstrate typical findings during mapping and ablation for SART.

## Conclusion

Sinoatrial reentry tachycardia is a relatively uncommon arrhythmia in the literature, but may be prone to underdiagnosis due to electrocardiographic similarity to sinus tachycardia and misinterpretation of symptoms as psychosomatic. Awareness of the diagnosis, and hence differentiation of SART from inappropriate sinus tachycardia, atrial tachycardia or non-cardiac diagnoses paves the way for adequate therapy. While medication (digoxin, verapamil and amiodarone) may be successful or even desirable under certain conditions, radiofrequency catheter ablation offers curative therapy in most cases with few adverse effects.

## Figures and Tables

**Table 1:**
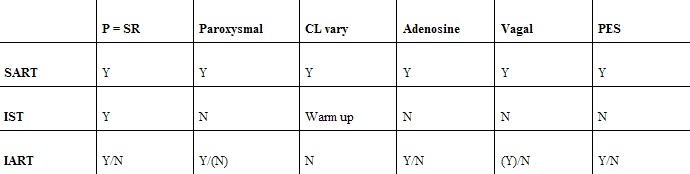
Differentiation between SART and the major differential diagnoses

SART = sinoatrial reentry tachycardia, IST = inappropriate sinus tachycardia, IART = intraatrial reentry tachycardia, "P = SR" denotes whether or not the P during tachycardia resembles sinus rhythm, CL = cycle length (referring to variation, in the case of IST warming up of the tachycardia), adenosine / vagal refers to whether or not adenosine or vagal manoeuvres terminate the tachycardia, PES = inducibility with programmed electrical stimulation?

## References

[R1] Barker PS, Wilson FN, Johnston FD (1943). The mechanism of auricular paroxysmal tachycardia. Am Heart J.

[R2] Han J, Malozzi AM, Moe GK (1968). Sino-atrial reciprocation in the isolated rabbit heart. Circ Res.

[R3]  Childers RW, Arnsdorf MF, de la Fuente DJ (1973). Sinus nodal echoes. Clinical case report and canine studies. Am J Cardiol.

[R4] Paulay KL, Varghese J, Damato AN (1973). Sinus node reentry. An in vivo demonstration in the dog. Circ Res.

[R5] Allessie MA, Bonke FIM (1979). Direct demonstration of sinus node reentry in the rabbit heart. Circ Res.

[R6] Paulay KL, Varghese PJ, Damato AN (1973). Atrial rhythms in response to an early premature atrial depolarization in man. Am Heart J.

[R7]  Narula OS (1974). Sinus node re-entry. A mechanism for supraventricular tachycardia. Circulation.

[R8] Cossu SF, Steinberg JS (1998). Supraventricular tachyarrhythmias involving the sinus node: clinical and electrophysiologic characteristics. Progr Cardiovasc Dis.

[R9] Wu D, Denes P, Amat-y-Leon F (1978). Clinical, electrocardiographic and electrophysiologic observations in patients with paroxysmal supraventricular tachycardia. Am J Cardiol.

[R10] Gomes JA, Hariman RJ, Kang PS (1985). Sustained symptomatic sinus node reentrant tachycardia: incidence, clinical significance, electrophysiologic observations and the effects of antiarrhythmic agents. J Am Coll Cardiol.

[R11] Gomes JA, Mehta D, Langan MN (1995). Sinus node reentrant tachycardia. PACE.

[R12] Wellens HJ, Bonke FIM (1978). Role of sinus node reentry in the genesis of sustained cardiac arrhythmias. The sinus node: structure, function and clinical relevance.

[R13] Weisfogel GM, Batsford WP, Paulay KL (1975). Sinus node re-entrant tachycardia in man. Am Heart J.

[R14] Kay GN, Chong F, Epstein AE (1993). Radiofrequency ablation for the treatment of primary atrial tachycardias. J Am Coll Cardiol.

[R15] Sperry RE, Ellenbogen KA, Wood MA (1993). Radiofrequency catheter ablation of sinus node re-entrant tachycardia. PACE.

[R16] Lesh MD, Van Hare GF, Epstein LM (1994). Radiofrequency catheter ablation of atrial arrhythmias. Results and mechanisms. Circulation.

[R17] Pahlajani DB, Miller RA, Serratto M (1975). Sinus node re-entry and sinus node tachycardia. Am Heart J.

[R18] Gillette PC (1976). The mechanisms of supraventricular tachycardia in children. Circulation.

[R19] Garson A, Gillette PC (1981). Electrophysiologic studies of supraventricular tachycardia in children. Am Heart J.

[R20] Blaufox AD, Numan M, Knick B (2001). Sinoatrial node re-entrant tachycardia in infants with congenital heart disease. Am J Cardiol.

[R21] Yee R, Guiraudon GM, Gardner MJ (1984). Refractory paroxysmal sinus tachycardia: management by subtotal right atrial exclusion. J Am Coll Cardiol.

[R22] Mischke K, Stellbrink C, Hanrath P (2001). Evidence of sinoatrial block as a curative mechanism in radiofrequency current ablation of inappropriate sinus tachycardia. J Cardiovasc Electrophysiol.

[R23] Simmers TA, Wittkampf FHM, Derksen R (2002). Isolation of residual sinus node during catheter ablation for the treatment of inappropriate sinus tachycardia. Neth Heart J.

[R24] Hendry PJ, Packer DL, Anstadt MP (1990). Surgical treatment of automatic atrial tachycardias. Ann Thorac Surg.

[R25] Kerr CR, Klein GG, Guiraudon GM (1988). Surgical therapy for sinoatrial re-entrant tachycardia. PACE.

[R26] Poty H, Saoudi N, Haissaguerre M (1996). Radiofrequency catheter ablation of atrial tachycardias. Am Heart J.

[R27] Farre J, Grande A, Martinell A, Brugada P, Wellens HJJ (1987). Atrial unipolar waveform analysis during retrograde conduction over left-sided accessory atrioventricular pathways. Cardiac arrhythmias: where to go from here?.

[R28] Haissaguerre M, Dartigues JF, Warin JF (1991). Electrogram patterns predictive of successful catheter ablation of accessory pathways. Value of unipolar recording mode. Circulation.

[R29] Simmers TA, Hauer RNW, Wever EFD (1994). Unipolar electrogram models for prediction of outcome in radiofrequency ablation of accessory pathways. PACE.

[R30] Delacretaz E, Soejima K, Gottipaty VK (2001). Single catheter determination of local electrogram prematurity using simultaneous unipolar and bipolar recordings to replace the surface ECG as a timing reference. PACE.

[R31] Villacastin J, Almendral J, Arenal A (2000). Usefulness of unipolar electrograms to detect isthmus block after radiofrequency ablation of typical atrial flutter. Circulation.

[R32] Man KC, Daoud EG, Knight BP (1997). Accuracy of the unipolar electrogram for identification of the site of origin of ventricular activation. J Cardiovasc Electrophysiol.

[R33] Tada H, Oral H, Knight BP (2002). Randomized comparison of bipolar versus unipolar plus bipolar recordings during segmental ostial ablation of pulmonary veins. J Cardiovas Electrophysiol.

[R34] Sanders WE, Sorrentino RA, Greenfield RA (1994). Catheter ablation of sinoatrial node reentrant tachycardia. J Am Coll Cardiol.

[R35] Ivanov MY, Evdokimov VP, Vlasenco VV (1998). Predictors of successful radiofrequency catheter ablation of sinoatrial tachycardia. PACE.

[R36] Simmers T, Sreeram N, Wittkampf F (2003). Catheter ablation of sinoatrial reentry tachycardia in a 2-month-old infant. Heart.

[R37] Leonelli FM, Pisano E, Requarth JA (2000). Frequency of superior vena cava syndrome following radiofrequency modification of the sinus node and its management. Am J Cardiol.

